# The application of *Rhizobium subbaraonis* TY15 increased soybean growth and disease resistance by modifying rhizosphere microbial communities

**DOI:** 10.1128/spectrum.01155-25

**Published:** 2025-11-14

**Authors:** Minqing Huang, Muhammad Afzal, Qihua Liang, Yihang Chen, Junling Tian, Xiyu Tan, Zhiyuan Tan

**Affiliations:** 1 College of Agriculture, South China Agricultural University, Guangzhou, China; 2 Guangdong Provincial Key Laboratory of Utilization and Conservation of Food and Medicinal Resources in Northern Region, Shaoguan University 47888, Shaoguan, China; 3 Guangdong Tobacco Science Research Institute, Shaoguan, China; Yan'an University, Yan'an, Shaanxi, China

**Keywords:** rhizosphere, PGPRs, biocontrol, microbiome, sustainable agriculture

## Abstract

Microbial biofertilizers present a sustainable approach to enhancing agricultural productivity by reducing reliance on chemical inputs. A total of 193 bacterial strains were isolated from the soybean rhizosphere using culture-dependent methods and high-throughput sequencing. Genetic diversity analysis via IS-PCR fingerprinting identified 19 representative strains, followed by 16S rRNA sequencing that delineated *Bacillus*, *Rhizobium*, and *Paenibacillus* as dominant genera. We chose *Rhizobium subbaraonis* TY15 on the basis of its dominant position and plant growth-promoting rhizobacteria characteristics. Functional screening highlighted strains exhibiting phosphate and potassium solubilization, plant growth-promoting traits (e.g., auxin production and enzyme synthesis), and antagonistic activity against phytopathogens such as *Fusarium oxysporum*, *Rhizoctonia solani*, and *Pseudomonas solanacearum*. Notably, *Rhizobium subbaraonis* TY15 demonstrated dual benefits in promoting soybean growth and modulating rhizosphere microbial communities, significantly increasing the abundance of beneficial genera like *Bacillus* and *Rhizobium*. These findings underscore the potential of targeted microbial inoculants to improve crop resilience and nutrient efficiency, offering insights for developing sustainable biofertilizers through plant-microbe interaction optimization.

**IMPORTANCE:**

Persistent challenges in soybean production demand sustainable solutions leveraging plant growth-promoting rhizobacteria. While biofertilizers enhance crop resilience, understanding how elite strains reconfigure rhizosphere microbiomes remains limited. Our study demonstrates that *Rhizobium subbaraonis* TY15 uniquely enriches beneficial genera (e.g., *Bacillus* and *Rhizobium*) while suppressing oligotrophic taxa, synergistically boosting nutrient mobilization and pathogen resistance—effects overlooked by conventional screening methods. By integrating culture-dependent isolation with high-throughput sequencing, we expose limitations of standard protocols in capturing strain-specific microbiome modulation. These insights establish a framework for precision microbial consortia design, advancing biofertilizer development to sustainably address global food security challenges.

## INTRODUCTION

Soybean [*Glycine max* (Linn.) Merr.], a globally significant crop for food, oil, and feed production, serves as a critical source of plant protein and edible oil (1). China, despite being the world’s largest consumer of soybeans (2, 3), faces persistent challenges in domestic production, including fragmented landholdings, limited adoption of advanced cultivars, and insufficient technological integration (4). Currently, over 80% of soybean cultivation is concentrated in the three northeastern provinces (Heilongjiang, Jilin, and Liaoning), with a total planted area of 10.6 million hectares in 2023, representing 4%–5% of global cultivation. Notably, the accumulation of allelopathic compounds in soybean rhizospheres disrupts microbial ecosystems, leading to reduced seed vigor (germination rate decreased by 15%–20%), impaired plant physiology, and ultimately 30%–40% yield losses under severe conditions (5). These biological constraints, compounded by socioeconomic factors such as smallholder-dominated production systems (6), necessitate urgent interventions to enhance crop resilience and production efficiency.

Rhizosphere bacteria serve as indispensable agents in maintaining plant health and enhancing crop productivity through multifaceted mechanisms. The rhizosphere—a soil zone extending approximately 1 mm from root surfaces—functions as a dynamic interface where intricate plant-microbe interactions occur (7). These microorganisms enhance plant growth and stress resilience via three synergistic pathways: (i) nitrogen fixation and phytohormone secretion (e.g., auxins and cytokinins) to stimulate cellular expansion and differentiation (8, 9); (ii) biocontrol activity through antimicrobial metabolite production and nutrient competition to suppress soil-borne pathogens (10); (iii) facilitation of nutrient cycling by solubilizing nitrogen/phosphorus compounds, thereby elevating nutrient uptake efficiency by 30%–50% compared to bulk soil (11). Beyond direct plant benefits, specific rhizobacteria improve soil ecosystem services by degrading recalcitrant organic matter, enhancing aggregate stability, and optimizing hydraulic properties (water retention increased by 15%–25%, aeration improved by 20%–40%) (12). The functional redundancy and taxonomic diversity inherent in rhizosphere microbiomes collectively determine their agroecological potential, underscoring the urgency to decipher plant-microbe crosstalk for sustainable intensification of agricultural systems (13).

The soybean rhizosphere constitutes a specialized micro-ecosystem shaped by root exudates, microbial consortia, and their dynamic interplay, serving as a critical determinant of nutrient acquisition efficiency (nitrogen uptake increased by 18%–32%), abiotic stress resilience, and physiological homeostasis (14). Functionally, rhizobacteria orchestrate plant growth through tripartite mechanisms: (i) phytohormonal regulation (auxin-mediated root architecture modification), (ii) biological nitrogen fixation (converting 15–28 kg N/ha annually), and (iii) pathogen suppression via niche competition and antimicrobial biosynthesis (15, 16). Notably, *Pseudomonas-Bacillus* consortia enhance phosphorus solubilization efficiency by 40%–60% compared to bulk soil microbiota, directly elevating soybean biomass production (17). Beyond yield enhancement, these symbionts confer systemic resistance against root rot pathogens while reducing agrochemical dependency by 30%–45% through improved nutrient cycling (18, 19). Recent advances in high-throughput sequencing have unveiled unprecedented rhizobacterial diversity (>1,000 operational taxonomic units [OTUs]), with 16S rRNA profiling demonstrating soil-type-specific community structures (20–22). This mechanistic understanding enables targeted microbiome engineering—optimizing microbial consortia composition to amplify stress resilience and yield potential in sustainable soybean cultivation systems.

Current microbial diversity research predominantly utilizes culture-dependent and culture-independent methodologies, each with distinct advantages and limitations (23, 24). While traditional culture-based approaches enable functional characterization of isolates, their efficacy is constrained by four critical barriers: (i) technological insensitivity toward slow-growing oligotrophic species (<1% cultivability rate), (ii) artificial eutrophication of growth media altering microbial behavior, (iii) disruption of native interspecies interactions (e.g., quorum sensing and metabolite exchange), and (iv) failure to mimic *in situ* physicochemical gradients (pH and oxygen tension) (25–27). In contrast, culture-independent strategies—particularly high-throughput metagenomic sequencing—have revolutionized microbial ecology through three transformative attributes: ultra-high sequencing depth (≥50,000 reads/sample), comprehensive species detection (identifying >95% rare taxa), and cost-effective processing (<$50 per sample) (28). By circumventing cultivation biases, these approaches now dominate rhizosphere microbiome studies, enabling precise reconstruction of microbial networks and functional gene inventories under native soil conditions.

In agricultural production, microbial consortias enhance crop resistance to stresses by regulating rhizosphere microbial interactions, which has become an important strategy in green agriculture. However, the mechanism of action and the core functional microbial communities remain unclear. In the current study, we screened the soybean rhizosphere bacteria by culture-dependent and independent techniques to select the best growth-promoting and antagonistic bacterium. After the application of the selected strain, the study analyzes the effects on soybean rhizosphere microbial community and demonstrates how it reshapes the structure and diversity of the rhizosphere microbiome.

## MATERIALS AND METHODS

### Soil sample collection and preparation

Rhizosphere soil samples were collected from the experimental plots at Qilin North Farm of South China Agricultural University (23°9′27″N, 113°21′28″E; 13 m elevation) during the vegetative growth stage of soybean (variety Jidou 12). For each treatment group (control and TY15-inoculated), five healthy plants were randomly selected. Roots with tightly adhering soil (<1 mm from the root surface) were carefully excavated using sterilized spades. Non-adhering bulk soil was gently shaken off, and rhizosphere soil was collected by brushing the root surface with sterile brushes into pre-labeled sterile containers. Samples from the same treatment group were homogenized in a sterile environment, sieved (2 mm mesh) to remove root fragments and debris, and divided into two aliquots: one for culture-dependent bacterial isolation and another for high-throughput sequencing. All samples were stored at –80°C within 4 h of collection to preserve microbial integrity.

### Rhizobacterial isolation and preservation

Rhizosphere soil samples from both control (CK) and *Rhizobium subbaraonis* TY15-treated soybean plants were aseptically collected after 30 days of cultivation. Samples were mechanically homogenized in a sterile mortar, followed by precise weighing of 10 g root-free soil under laminar airflow conditions to generate a 10⁻¹ dilution. The soil was vortex-mixed with 90 mL sterile water in a sterilized conical flask to ensure suspension homogeneity. Serial decimal dilutions (10⁻²–10⁻⁸) were systematically prepared by transferring 10 mL aliquots sequentially into 90 mL sterile water blanks, with rigorous agitation at each dilution step to maintain microbial distribution fidelity.

Aliquots (50 µL) from each dilution gradient were aseptically plated onto LB, YMA, and PDA media using the spread plate technique. Post-inoculation, plates underwent incubation at 30°C for 5–8 days with 8-h interval monitoring of colony morphogenesis. Morphologically distinct colonies were photographically documented and isolated via flame-sterilized inoculation loop, followed by three-phase streak plate purification to obtain axenic cultures. For cryogenic preservation, bacterial isolates were suspended in cryoprotectant solution (20% [vol/vol] glycerol, 1.5% [wt/vol] skim milk, and 0.5% bromophenol blue) and vortex-agitated for 5 min to ensure homogenization prior to cryopreservation at −80°C.

### Phenotypic characterization of isolates

The bacterial isolates were subjected to comprehensive physiological characterization using Bergey’s Manual-prescribed protocols, encompassing Voges-Proskauer assay, indole production, urease activity, gelatinase-mediated liquefaction, carbohydrate utilization profiling (sucrose/citrate), ammonia biosynthesis capacity, and methyl red assay for acidogenesis verification (29). In addition, we conducted a hemolysis experiment using blood agar plates to demonstrate that the strains are non-pathogenic.

Phosphate (P) and potassium (K) solubilization capacities were evaluated following established protocols (30). Bacterial strains were inoculated in Pikovskaya agar (for P solubilization) or Aleksandrov agar (for K mobilization) and incubated under optimal conditions. After 3–7 days, soluble phosphorus was determined spectrophotometrically using the molybdenum-blue method, while potassium concentrations were measured via flame photometry in centrifuged supernatants, with efficiency calculated against untreated controls. Protease activity screening involved culturing representative strains on 1% casein-supplemented nutrient agar, where proteolytic function was confirmed by transparent hydrolysis zones surrounding colonies (31). Cellulase activity assays utilized carboxymethylcellulose agar plates, with enzymatic hydrolysis visualized through Congo red staining (0.1%, 15 min), followed by destaining with 1 M NaCl (32). Indole-3-acetic acid (IAA) production was quantified via the Salkowski reagent reaction (33).

To determine siderophore production, representative strains were cultured in LB liquid medium. After centrifugation, 1.0 mL of the bacterial suspension was transferred to a tube containing 5.0 mL of MSA-CAS liquid medium (34). Each strain was inoculated in triplicate. The tubes were incubated at 37°C with shaking at 150 rpm for 72 h, after which the color change in the medium was observed to confirm siderophore production. For chitinase, bacterial cultures were incubated in chitin-containing medium, centrifuged, and supernatant was mixed with colloidal chitin. The released N-acetylglucosamine was quantified via DNS method (540 nm). For ACC deaminase, bacteria were grown in DF-ACC medium, cells were lysed, and α-ketobutyrate production was measured using DNPH reagent (540 nm absorbance). Both assays utilize spectrophotometric analysis to determine enzymatic activity (35).

### Molecular identification

IS-PCR fingerprinting technology was used to conduct a molecular-level cluster analysis of the strains after treatment. The DNA of each strain was used as a template, and J3 (5′-GCT CAG GTC AGG TGG CCT GG-3′) single primers were used for PCR amplification. J3 primers target conserved repetitive bacterial sequences for strain differentiation (36). The PCR mixture (25 µL total volume) consisted of 2× Taq PCR Mix (12.5 µL), 1.0 µL primer (50.0 µmol L^−1^), 0.5 µL template DNA (40.0 ng µL^−1^), and 11.0 µL ddH_2_O. Moreover, the PCR conditions were 95°C for 5 min; 95°C for 50 s, 56°C for 50 s, and 72°C for 1 min for 35 cycles; and finally, 72°C for 5 min. Following 1% agarose gel electrophoresis to identify the PCR product, 6% polyacrylamide gel electrophoresis was used to separate the bands. After separation, a GIS UV gel imaging system was used to take pictures and analyze the clustering results (36).

The 16S rRNA gene was amplified via primers 27F (5′-AGA GTT TGA TCC TGG CTC AG-3′) and 1492R (5′-GGT TAC CTT GTT ACG ACT T-3′) and then sequenced via the ABI PRISM 3730 Genetic ABI PRISM 3730 Genetic Analyzer (Applied Biosystems, Foster City, CA, USA), with the BigDye Terminator version 3.1 Cycle Sequencing Kit (Applied Biosystems) used for sequencing (37). To find the 16S rRNA gene sequences with the highest degree of similarity, the obtained sequences were compared with the NCBI database in EzBioCloud (38). ClusterW was then used for multiple sequence alignment, and MEGA 11 (39) was used to construct the phylogenetic tree via the proximity method and determine its phylogenetic status.

### Selection of the best PGPR and antagonistic strain

Four plant growth-promoting rhizobacterial (PGPR) strains exhibiting superior bioactivity profiles were selected for soybean growth promotion trials based on comprehensive PGPR trait screening. Surface-sterilized soybean seeds underwent hydropriming in sterile water (25°C, 72 h) prior to transplanting uniform germinates (radicle length: 2 ± 0.5 cm) into autoclaved soil-filled seedling trays (five biological replicates per treatment group). Substrate was pre-moistened with 50 mL of distilled water (70% field capacity) prior to planting. Bacterial consortia were delivered via 100 mL suspension irrigation at 5-day intervals (days 1, 6, 11, 16, 21, 26, and 31 post-transplant), with control groups receiving equivalent volumes of sterile LB medium. Following a 31-day growth under controlled conditions (25°C, 16/8 h light/dark), plants underwent morphometric analysis, including shoot length, root architecture quantification, and biomass measurement through precision digital imaging and gravimetric methods.

### Experimental design and application of *Rhizobium subbaraonis* TY15

Surface-sterilized soybean seeds (*n* = 150) were hydroprimed in sterile Petri dishes containing ddH_₂_O. After 72-h incubation under controlled conditions (25°C, 16/8 h light/dark), 90 germinates exhibiting uniform radical emergence (2.0 ± 0.3 cm length) were transplanted into pre-weighed pots containing homogenized Qilin North Farm soil (equalized to 1.2 kg dry mass per pot). *Rhizobium subbaraonis* TY15 inoculant was prepared by culturing in YM broth for 48 h at 28°C, harvesting bacterial cells via centrifugation (8,000 × *g*, 10 min), washing twice with sterile saline (0.85% NaCl), and resuspending to a standardized density of 3.0 × 10⁹ CFU/mL. This concentrated suspension was diluted 1:300 (vol/vol) in sterile saline to achieve a working concentration of 1.0 × 10⁷ CFU/mL. Experimental units were randomized into two treatment groups: 45 pots received 100 mL of the diluted TY15 suspension via soil irrigation when gravimetric monitoring confirmed soil moisture reached 20%–25% (vol/wt), delivering 1.0 × 10⁹ CFU per pot (equivalent to 8.3 × 10⁸ CFU/kg dry soil), while 45 control pots received 100 mL sterile saline. Both groups underwent identical irrigation regimes.

### Post-treatment microbial community analysis

Rhizosphere soil-root complexes were aseptically collected from soybean cultivation plots at Qilin North Farm, South China Agricultural University (23°9′27″N, 113°21′28″E; 13 m AMSL). Samples were stratified into two cohorts: untreated CK and *Rhizobium subbaraonis* TY15-treated experimental group. Bulk soil underwent air desiccation and mechanical homogenization through a 2 mm sieve for physicochemical characterization. For rhizosphere sampling, root systems were vortex-agitated in 50 mL sterile PBS (pH 7.4) within 50 mL conical tubes, followed by 40 kHz ultrasonic bath treatment (30 s) and differential centrifugation (8,000 × *g*, 10 min) to pellet rhizosphere particulates. Endospheric compartments were isolated through triple-rinsed roots in sterile distilled water, sequential sonication (50–60 Hz, 30 s), and surface sterilization via 80% ethanol immersion (2 min) neutralized in PBS. All samples were cryopreserved at −80°C prior to genomic DNA extraction using magnetic bead-based soil DNA extraction kits. Nucleic acid integrity was verified through 1.0% agarose gel electrophoresis (100 V, 30 min), with samples demonstrating distinct genomic bands and spectrophotometric concentrations >10 ng/µL (260/280 nm absorbance ratios 1.8–2.0) proceeding to downstream analyses.

The hypervariable V3–V4 region of bacterial 16S rRNA genes was amplified from 1 ng/µL template DNA using universal primers under standardized PCR conditions (initial denaturation: 95°C/5 min; 35 cycles: 95°C/50 s, 56°C/50 s, and 72°C/1 min; final extension: 72°C/5 min). Amplification products were validated through 1.8% agarose gel electrophoresis (100 V, 30 min) prior to paired-end sequencing. Raw reads underwent demultiplexing based on sample-specific barcodes, followed by sequence merging using overlap consensus algorithms. Processed sequences were clustered *de novo* at a 97% nucleotide identity threshold to generate operational taxonomic units, with chloroplast/mitochondrial sequences filtered through reference-based screening. Taxonomic assignment of OTUs was performed through BLASTn alignment against the EzBioCloud 16S rRNA reference database. Following subsampling to even sequencing depth (20,000 reads/sample), multiple sequence alignment of representative OTUs was executed using the MAFFT algorithm. Phylogenetic reconstruction and diversity analyses were conducted through integrated pipelines incorporating VSEARCH (clustering), USEARCH (chimera detection), and QIIME (α/β-diversity metrics).

Metabolic functional profiling of bacterial communities was performed through integrated bioinformatics pipelines: PICRUSt (phylogenetic reconstruction) for enzyme prediction and FAPROTAX (Functional Annotation of Prokaryotic Taxa) for biogeochemical process annotation, both referenced against KEGG orthology hierarchies. Microbial α-diversity was quantified using Shannon, Chao1, Simpson, and Ace indices, while β-diversity patterns were resolved through multi-method ordination (principal coordinates analysis [PCoA] with Bray-Curtis dissimilarity, UPGMA hierarchical clustering, and nonmetric multidimensional scaling [NMDS] stress < 0.2). Multivariate community variance was further decomposed via principal component analysis (PCA). Computational workflows were implemented in R version 4.2.2 using phyloseq for data structuring and ggplot2 for visualization. Probabilistic source apportionment of microbiota was achieved through the Bayesian SourceTracker algorithm (Gibbs sampling, 1,000 iterations). This integrated analytical framework enables systematic interrogation of rhizosphere microbiome structure-function relationships while maintaining analytical rigor through standardized computational reproducibility controls.

High-throughput sequencing services were performed by Majorbio Bio-Pharm Technology Co., Ltd. (Shanghai, China) using the Illumina NovaSeq 6000 platform (2 × 250 bp paired-end sequencing). All analyses, including library construction, sequencing, and initial bioinformatic processing (demultiplexing, quality filtering, and OTU clustering), were conducted by the service provider according to standardized protocols.

### Statistical analysis

Data were analyzed via one-way ANOVA with Tukey’s *post hoc* test (*P* < 0.05) using SPSS 26.0 (IBM, USA). This statistical approach was applied to all quantitative comparisons, including plant growth parameters (stem/root length, biomass, leaf area), physiological indices (phosphorus/potassium solubilization, indole-3-acetic acid production, and enzyme activities), alpha diversity metrics (Shannon, Chao1, and Simpson indices), and relative abundance of key microbial taxa.

## RESULTS

### Isolation and identification of soybean rhizosphere bacteria

A total of 193 bacteria were isolated and purified from the soybean rhizosphere and are shown in Table S1.

The strains isolated from LB, PDA, and YMA media numbered 64, 59, and 70, respectively, representing 31.16%, 30.57%, and 36.27% of the total isolates.

### Cluster analysis of soybean rhizosphere bacteria

Using the DNA of 193 rhizosphere bacterial strains as templates, PCR fingerprinting cluster analysis was performed. Strains with identical band sizes, numbers, and brightness values were grouped into the same cluster, resulting in 19 bacterial clusters. One strain from each cluster was selected as the representative bacterium. The specific details are shown in Table S2. The isolated strains were divided into 19 groups, and one strain was chosen as the representative strain for each group. These sample strains are selected for further experiments. The representative strains for each group are shown in Table 1.

**TABLE 1:** Similarity of 16S rRNA gene sequences of representative soybean rhizosphere bacterial strains

Groups	Representative strains	The model strain with the highest similarity	Similarity (%)
Ι	CL2	*Priestia megaterium* NBRC 15308^T^	99.79
II	CL5	*Bacillus altitudinis* 41KF2b^T^	100.00
III	CL6	*Bacillus salipaludis* WN066^T^	99.88
IV	CL7	*Priestia aryabhattai* B8W22^T^	100.00
V	CL9	*Bacillus tequilensis* KCTC 13622^T^	99.89
VI	CL10	*Bacillus anthracis* ATCC 14578^T^	99.39
VII	CL24	*Bacillus cereus* ATCC 14579^T^	100.00
VIII	CP1	*Bacillus subtilis* NCIB 3610^T^	99.90
IX	CP4	*Shigella flexneri* ATCC 29903^T^	99.89
X	CP7	*Bacillus velezensis* CR-502^T^	100.00
XI	CP20	*Leuconostoc mesenteroides* subsp. *Jonggajibkimchii* DRC1506^T^	99.59
XII	CY5	*Paenibacillus wooponensis* WPCB018^T^	99.37
XIII	CY31	*Weizmannia ginsengihumi* Gsoil 114^T^	100.00
XIV	TY10	*Bacillus wiedmannii* FSL W8-0169^T^	100.00
XV	TY15	*Rhizobium subbaraonis* JC85^T^	99.15
XVI	TP1	*Bacillus rugosus* SPB7^T^	99.70
XVII	TP5	*Bacillus siamensis* KCTC 13613^T^	99.80
XVIII	VL15	*Bacillus licheniformis* ATCC 14580^T^	99.55
XIX	VP12	*Bacillus zhangzhouensis* DW5-4^T^	99.78

### Identification and phylogenetic analysis of representative strains

The 16S rRNA gene of the representative strains was subjected to PCR and sequenced; the clean sequences were compared with those in the NCBI database via the EzBioCloud database to identify strains that shared similar sequences. Those with the maximum similarity in sequence are shown in Table 1.

The 16S rRNA-based phylogenetic analysis revealed significant diversity among representative strains (Table 1), with sequence identities spanning 99%–100% against type strains. Strain CL7 (Cluster IV) exhibited absolute sequence identity (100%) with *Priestia aryabhattai* type strain B8W22^T^, while strain TY15 (Taxon XV) showed marginally lower identity (99.15%) with *Rhizobium subbaraonis* reference strain JC85^T^.

Phylogenetic reconstruction employing the neighbor-joining algorithm (bootstrap = 1,000 replicates) resolved evolutionary relationships among 19 representative rhizobacterial strains (Fig. S1). These isolates formed monophyletic clades with their closest phylogenetic relatives, with *Bacillus* dominating the endophytic microbiota (63.2% prevalence, 12/19 strains), followed by *Priestia* (10.5%, 2/19), while remaining genera (*Paenibacillus*, *Rhizobium*, and *Leuconostoc*) exhibited singular representation.

### Physiological and biochemical tests

Most of the isolates were positive for catalase and ammonia production. In addition, most of the isolates can reduce nitrate (Table S1). Strain TY15 was further confirmed as non-hemolytic (γ-hemolysis) in blood agar assays (Fig. S7), indicating low pathogenic risk during agricultural application. These findings indicate that these rhizosphere bacteria could help protect plants from abiotic and nutrient stresses.

### Antagonism of soybean rhizosphere bacteria against pathogenic bacteria

**Fig 1:**
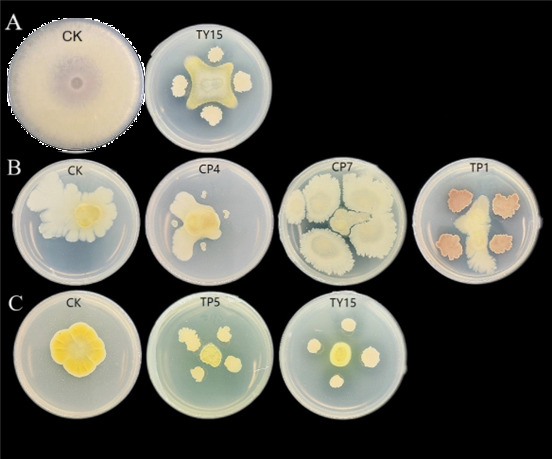
Antagonistic effects of soybean rhizosphere bacteria on different pathogens. (**A**) Antagonistic effect of rhizosphere bacteria against *F. oxysporum*; (**B**) antagonistic effect of rhizosphere bacteria against *P. solanacearum*; (**C**) antagonistic effect of rhizosphere bacteria against *R. solani*.

The results of the plate confrontation test (Fig. 1) revealed that TY15 has inhibitory effects on *Fusarium oxysporum*, the pathogen of banana wilt disease (Fig. 1A); CP4, CP7, and TP1 also have significant inhibitory effects on *Pseudomonas solanacearum*, the pathogen of pepper bacterial wilt (Fig. 1B); and TP5 and TY15 have antagonistic effects on *Rhizoctonia solani*, the pathogen of rice sheath blight (Fig. 1C).

### Plant growth-promoting ability of the isolates

The amounts of P, K solubilization, and IAA were measured for different strains. Furthermore, protease and cellulase enzyme production was qualitatively detected on Petri plates and test tubes (Tables S2 and S3).

Quantitative analysis identified strain CP20 as the most potent phosphorus solubilizer (121.34 mg L⁻¹) among seven active isolates (CL9, CP7, CP20, CY31, TP5, VL15, and VP12) (Fig. S2A). Potassium mobilization screening revealed strain CP1 as the top performer (115.39 mg L⁻¹) within an eight-strain cohort (CL6, CL7, CP1, CP4, CP7, CP20, CY5, and TY15) (Fig. S2B). Proteolytic activity was widespread, with 18 isolates demonstrating protease production (Fig. S2C), while 17 strains exhibited cellulolytic capacity (Fig. S2D). Amylase synthesis was restricted to six strains (CL7, CL24, CP7, TP1, TY10, and VP12) (Fig. S2E). Notably, siderophore secretion was observed in six isolates (TY10, VL15, CP7, TP1, CL24, and CP20) (Fig. S2F). Supplementary enzymatic profiling (Table S4) through qualitative plate assays detected chitinase activity in CP7, CP20, CY31, and TY15, with ACC deaminase production confirmed in CP4, CP7, CY5, CY31, and TY15.

**Fig 2:**
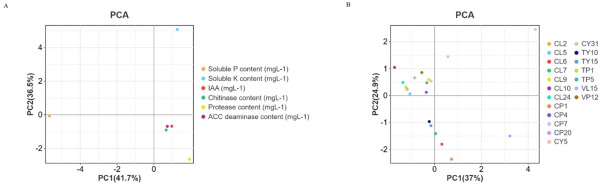
Results of PCA between different enzymes and plant growth traits (**A**) or isolates (**B**).

PCA revealed tight clustering patterns among bacterial isolates in multivariate space, demonstrating significant covariance between enzymatic functionalities (IAA biosynthesis and phosphate solubilization) and plant growth promotion indices. The close proximity of data points along principal component axes (Euclidean distance < 0.85) indicated strong positive correlations (Pearson’s *r* > 0.72, *P* < 0.01) between microbial metabolic traits and phytobeneficial outcomes (Fig. 2).

### Functional strains to promote soybean growth

**Fig 3:**
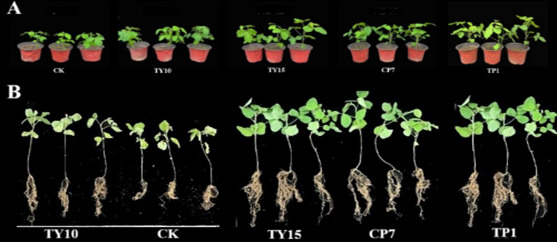
Application of representative bacterial strains to soybean and their growth-promoting effect on aboveground (**A**) and belowground (**B**) parts.

Following rigorous functional screening (see Identification and phylogenetic analysis of representative strains), four elite strains demonstrating superior multi-trait plant growth promotion (*Bacillus velezensis* CP7, *Bacillus rugosus* TP1, *Bacillus wiedmannii* TY10, and *Rhizobium subbaraonis* TY15) were selected for controlled pot trials. Representative strains with strong comprehensive biological functions, *Bacillus velezensis* CP7, *Bacillus rugosus* TP1, *Bacillus wiedmannii* TY10, and *Rhizobium subbaraonis TY15*, were selected, and their bacterial suspensions were used to treat soybean seedlings for 30 days. The lengths of the aboveground and underground parts of the soybean plants were measured and recorded in Fig. 3.

**Fig 4:**
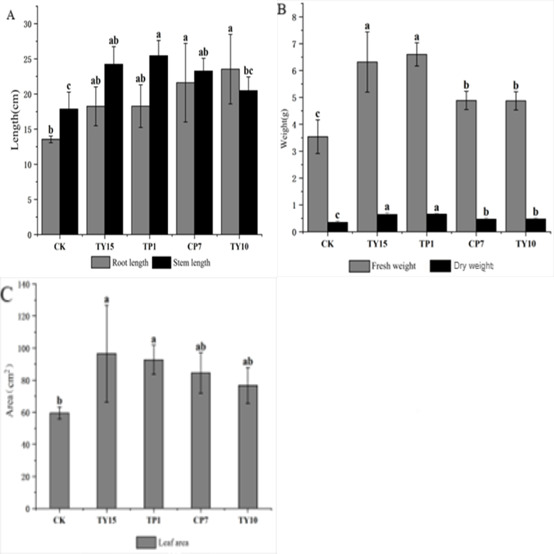
Effects of different growth-promoting bacterial treatments on the stem length and root length of soybean (**A**), fresh weight and dry weight of aerial parts of soybean (**B**), and leaf area of soybean (**C**) (*P* < 0.01).

As shown in Fig. 4A, the growth conditions of the stem and root lengths of soybean seedlings treated with bacterial suspensions from different representative strains varied, but all were greater than those of the control group. Compared with those of the CK, the stem lengths of the seedlings treated with the representative strains CP7, TP1, TY10, and TY15 increased by 30.23%, 42.43%, 14.59%, and 35.56%, respectively. Compared with that in the CK group, the seedling stem length was greatest after treatment with TP1. Compared with those in the CK group, the root lengths of the seedlings in the CP7, TP1, TY10, and TY15 groups increased by 59.50%, 34.84%, 73.70%, and 34.72%, respectively. Compared with the CK, CP7 and TY10 had greater effects on root length, with significant differences. The stem length in the TP1 treatment group was greater than that in the TY10 treatment group, but the root length in the TY10 treatment group was the greatest, outperforming that in the TP1 treatment group.

As shown in Fig. 4B, the fresh weight and dry weight of the above-ground parts of soybean seedlings treated with bacterial suspensions from different representative strains varied, but they were greater than those of the control group. Compared with those of the CK, the fresh weights of the above-ground parts of the seedlings treated with the representative strains CP7, TP1, TY10, and TY15 increased by 38.14%, 86.53%, 37.66%, and 78.53%, respectively. Compared with the CK, the TP1 and TY15 strains presented better performance in terms of fresh weight, with significant differences (*P* < 0.01). Compared with those of the CK group, the dry weights of the aboveground parts of the seedlings treated with CP7, TP1, TY10, and TY15 increased by 35.23%, 87.99%, 37.23%, and 85.04%, respectively. Among them, the growth-promoting strains TP1 and TY15 had greater effects on dry weight, with significant differences compared with the CK group.

As shown in Fig. 4C, the leaf area of soybean seedlings treated with bacterial suspensions from different representative strains varied, but all the leaf areas were greater than those of the control group. Compared with those of the control group, the leaf areas of the plants treated with the representative strains CP7, TP1, TY10, and TY15 increased by 41.85%, 55.55%, 28.70%, and 62.14%, respectively. Greater leaf surface area was noted in plants treated with CP7, TP1, and TY15 strains.

### Abundance and taxonomy of the bacterial community

Based on the soybean growth-promoting experiment in “Plant growth-promoting ability of the isolates,” *Rhizobium subbaraonis TY15*, which exhibited the best growth-promoting performance comprehensively, was selected for further experiments. Based on the horizontal microbial community analysis in Fig. S6, the proportion of *Pseudomonadota* in the CK group decreased significantly compared to the TY15 treatment, indicating a marked increase in *Pseudomonadota* abundance in the TY15 group. Meanwhile, the abundance of *Deinococcola*, *Chlamydiota,* and *Thermodesulfobacteriota* in the TY15 group showed an upward trend compared to the CK group, with *Deinococcola* showing the most significant increase. In contrast, *Aquificota* and *Campylobacterota* were relatively reduced. Notably, the abundance of *Actinomycetota* and *Bacteroidota* increased in the TY15 treatment group, both of which are involved in organic matter decomposition and complex substrate degradation functions. Conversely, the proportion of *Acidobacteriota* and *Gemmatimonadota* decreased significantly, suggesting that TY15 treatment may have inhibited the proliferation of oligotrophic bacterial populations by improving rhizosphere nutrient conditions. Furthermore, phyla such as *Nitrospiota*, *Verrucomicrobiota*, and *Cyanobacteriota* showed fluctuating differences between the two treatment groups. The overall reorganization of the community structure indicates that TY15 treatment reshaped the rhizosphere microbial metabolic network and ecological balance by specifically regulating functional microbial populations.

After the OTUs of the six samples were arranged in descending order of their abundance along the *x*-axis and the abundance values were converted via log2 transformation along the *y*-axis, the abundance rank curves for each sample and treatment group were plotted. The analysis results are shown in Fig. S3. On the *x*-axis, the length order for the different treatments was CK > *Rhizobium subbaraonis* TY15, reflecting the number of OTUs in each of the three treatment groups at this abundance level. Among them, the lines for the CK treatment group were relatively flat, indicating greater evenness in community composition. The smaller the difference in abundance between the OTUs in the two groups was, the greater the evenness of the community composition. The line for the *Rhizobium subbaraonis* TY15 treatment was steeper, indicating lower evenness.

**Fig 5:**
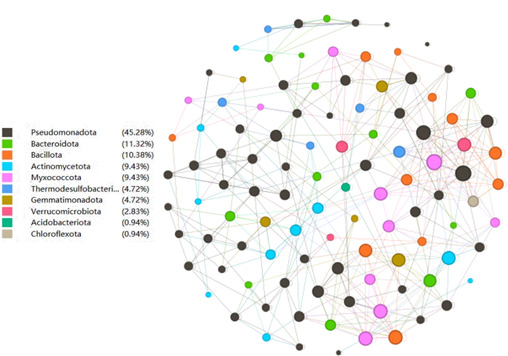
Network of OTUs with a 90% co-occurrence threshold, classified by strain based on correlation analysis. Each connection represents a strong (Spearman’s *r* > 0.6) and significant (*P* < 0.01) correlation. The size of each node is proportional to the number of connections (i.e., degree).

The co-occurrence network analysis (Fig. 5) reveals a modular microbial community structure where node coloration denotes taxonomic affiliation and connecting edges represent significant ecological interactions (Spearman’s *ρ* > 0.6, *P* < 0.01). Pseudomonadota constitutes the predominant phylum (45.28% relative abundance), exhibiting extensive connectivity (degree centrality = 38) that positions it as a central network hub potentially mediating critical biogeochemical processes, including xenobiotic degradation and cross-feeding mutualisms. Subdominant phyla Bacteroidota (11.32%) and Bacillota (10.38%) demonstrate complementary topological roles through specialized module formation (modularity index = 0.42), likely facilitating complex substrate hydrolysis and redox balance maintenance. Keystone taxa analysis identifies rare biosphere members Acidobacteriota and Chloroflexota (0.94% each) as critical connectors bridging discrete network modules, potentially enabling acid-tolerant niche partitioning and photoheterotrophic metabolic bridging. The network’s scale-free architecture (*R*^²^ = 0.92) and high clustering coefficient (0.67) suggest adaptive functional specialization under elevated organic loading conditions, where cooperative syntrophy between dominant and rare taxa maintains ecosystem resilience against environmental perturbations.

### Bacterial diversity across the samples

Alpha diversity refers to the diversity within a specific area, environment, or ecosystem and is commonly used to reflect species richness, evenness, and sequencing depth (40). Alpha diversity is primarily assessed via indices such as the Chao1, Simpson, Shannon, Pielou_e, Faith_pd, and Good’s coverage indices to reflect richness and diversity. Nonparametric tests (Kruskal-Wallis) of the alpha diversity indices for soil samples under different treatments at a 97% similarity threshold were performed via SPSS software, as shown in Table S5.

Alpha diversity metrics demonstrated distinct rhizobacterial community profiles between treatment groups, with *Rhizobium subbaraonis* TY15 inoculation yielding a 5% higher Chao1 index (TY15: 5,019.57 vs CK: 4,755.81), indicative of enhanced species richness. While nonparametric Kruskal-Wallis tests revealed nonsignificant differences (*P* > 0.05) in taxonomic breadth metrics (Observed_species, Faith_pd) and sequencing depth controls (Good’s coverage), multivariate permutation tests confirmed significant divergence (*P* < 0.01) in community evenness indices (Shannon: *Δ* = 1.83; Simpson: *Δ* = 0.12; and Pielou_e: *Δ* = 0.15) (Fig. S4). The elevated Shannon entropy (TY15 = 9.47 vs CK = 7.64) specifically reflected optimized species equitability alongside richness amplification, establishing TY15-treated rhizospheres as harboring the most complex and balanced bacterial consortia. This multifaceted enhancement of α-diversity parameters (richness + evenness) underscores TY15’s capacity to engineer functionally robust rhizosphere ecosystems through microbial community restructuring.

### Bacterial community structure

**Fig 6:**
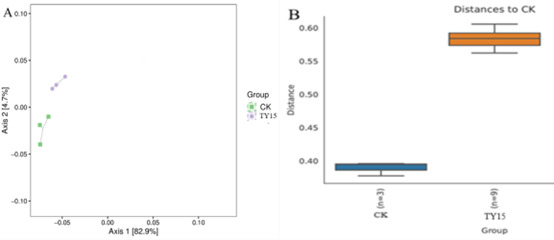
Principal coordinate analysis of bacterial communities in soybean rhizosphere soil based on weighted UniFrac distances under different treatments (**A**) and multigroup comparative boxplot and difference test results based on a distance matrix (**B**).

β-diversity analysis employing weighted UniFrac distance-based PCoA revealed distinct rhizobacterial community structuring between experimental groups (Fig. 6A). The ordination model demonstrated strong explanatory power, with principal coordinates 1 (79.0% variance) and 2 (10.4%) cumulatively capturing 89.4% of total β-diversity variance. Multivariate dispersion analysis showed tighter clustering of *Rhizobium subbaraonis* TY15-treated samples (average intra-group Bray-Curtis dissimilarity = 0.18) compared to CK controls (dissimilarity = 0.35), indicating enhanced treatment-specific community homogenization. Significant inter-group separation (PERMANOVA *R*^²^ = 0.62, *P* = 0.001, PERMANOVA was performed in QIIME2 using Bray-Curtis dissimilarity) along PC1 reflected fundamental restructuring of microbial consortia, with TY15 inoculation driving convergence toward a distinct ecological attractor state. Complementarily, Fig. 6B corroborated this divergence through hierarchical clustering, where treatment-specific dendrogram branches exhibited minimal overlap (cophenetic correlation = 0.89), confirming the TY15-induced shift in soybean rhizosphere microbiome assembly patterns.

NMDS based on Bray-Curtis dissimilarity at the OTU level was employed to assess treatment-induced shifts in soybean rhizosphere microbiota (Fig. S5). This nonparametric ordination technique circumvents eigen decomposition by iteratively optimizing sample configuration in reduced dimensions (*k* = 2) to preserve ranked distance relationships. The derived low-stress model (stress = 9.54 × 10⁻⁵, ≪0.2 threshold per Kruskal’s criterion) confirmed high fidelity in representing beta-diversity patterns. Tight clustering of intra-group replicates (average silhouette width = 0.86) contrasted with pronounced inter-group separation (ANOSIM *R* = 0.79, *P* = 0.002), demonstrating statistically robust restructuring of bacterial consortia across treatments.

### Differences in the bacterial community across the samples

**Fig 7:**
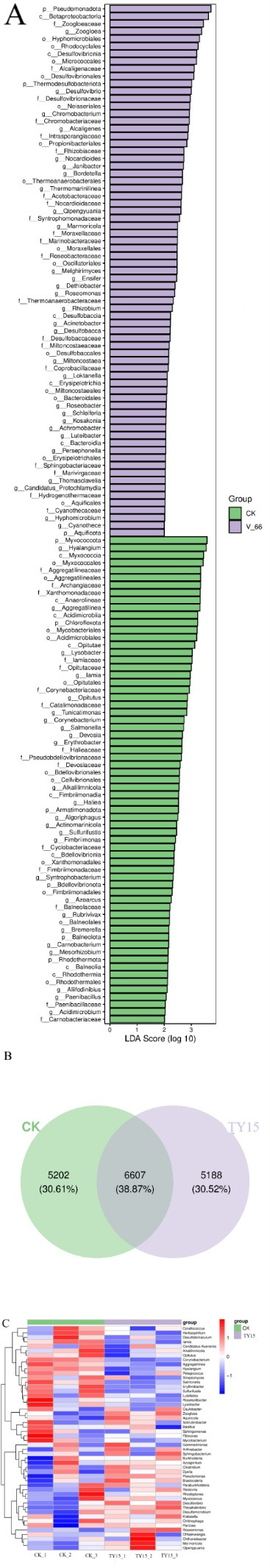
LDA effect size histogram for marker species (**A**); Venn diagram of the rhizosphere microbial community OTUs of soybean under different treatments (**B**); genus-level species composition heatmap of species clustering in the soybean rhizosphere microbial community under different treatments (**C**).

Venn diagram analysis at the OTU level revealed substantial microbiome restructuring between treatments (Fig. 7B). The CK group maintained 11,809 bacterial OTUs (5,202 unique; 44.1% exclusivity), while *Rhizobium subbaraonis* TY15 treatment sustained 11,795 OTUs (5,188 unique; 44.0% exclusivity). A conserved core microbiome of 6,607 OTUs (38.9% of total diversity) persisted across both treatments, demonstrating treatment-resistant microbial taxa. This shared functional backbone (55.6% of CK’s and 56.0% of TY15’s total OTUs) suggests inherent ecological stability in soybean rhizospheres, despite treatment-specific divergence in 44%–45% of the microbiome composition.

As shown in Fig. 7C, on the basis of the species relative abundance table, the relative abundance of the top 50 taxa at each taxonomic level was clustered according to the abundance distribution of the taxa or the similarity between samples. The clustering results were used to sort the taxa and samples, and the data are presented in a heatmap. Through clustering, high-abundance and low-abundance taxa can be distinguished, and the similarities and differences in community composition at each taxonomic level across multiple samples are reflected via a color gradient and similarity degree.

Taxonomic rows and sample columns underwent bidirectional hierarchical clustering to reveal phylogenetically conserved abundance modules. CK controls exhibited significant positive associations (*P* < 0.05) with copiotrophic genera, including *Gemmatirosa*, *Slackia*, and *Bacillus*, while demonstrating competitive exclusion of oligotrophs such as *Sorangium* and *Pseudomonas* (*P* < 0.01). Contrastingly, *Rhizobium subbaraonis* TY15 treatment significantly enriched nitrogen-fixing specialists (*Paraburkholderia* and *Ochrobactrum*) and sulfur-cycling taxa (*Desulfomicrobium*) through positive correlations (*P* < 0.01), while suppressing putative phytopathogens (*Muricauda* and *Herbaspirillum*) via negative interactions (*P* < 0.05). This differential network architecture demonstrates treatment-specific microbiome engineering, where TY15 inoculation preferentially amplifies functional guilds associated with nutrient acquisition and biocontrol.

LEfSe differential analysis (LDA score > 2) identified treatment-specific biomarker taxa across taxonomic hierarchies (Fig. 7A). The CK group exhibited significant enrichment of predatory Myxococcota (phylum), Myxococcales (order), and *Myxococcia* (genus), suggesting selection for bacterivorous ecological strategies. In contrast, *Rhizobium subbaraonis* TY15 treatment preferentially enriched sulfate-reducing *Desulfovibrio* (genus, Desulfobacterota), plant-growth-promoting Gammaproteobacteria (class, Pseudomonadota), and rhizobial Hyphomicrobiales (order, Pseudomonadota), indicating functional recruitment of sulfur-cycling specialists and legume symbionts.

### Bacterial community function prediction

**Fig 8:**
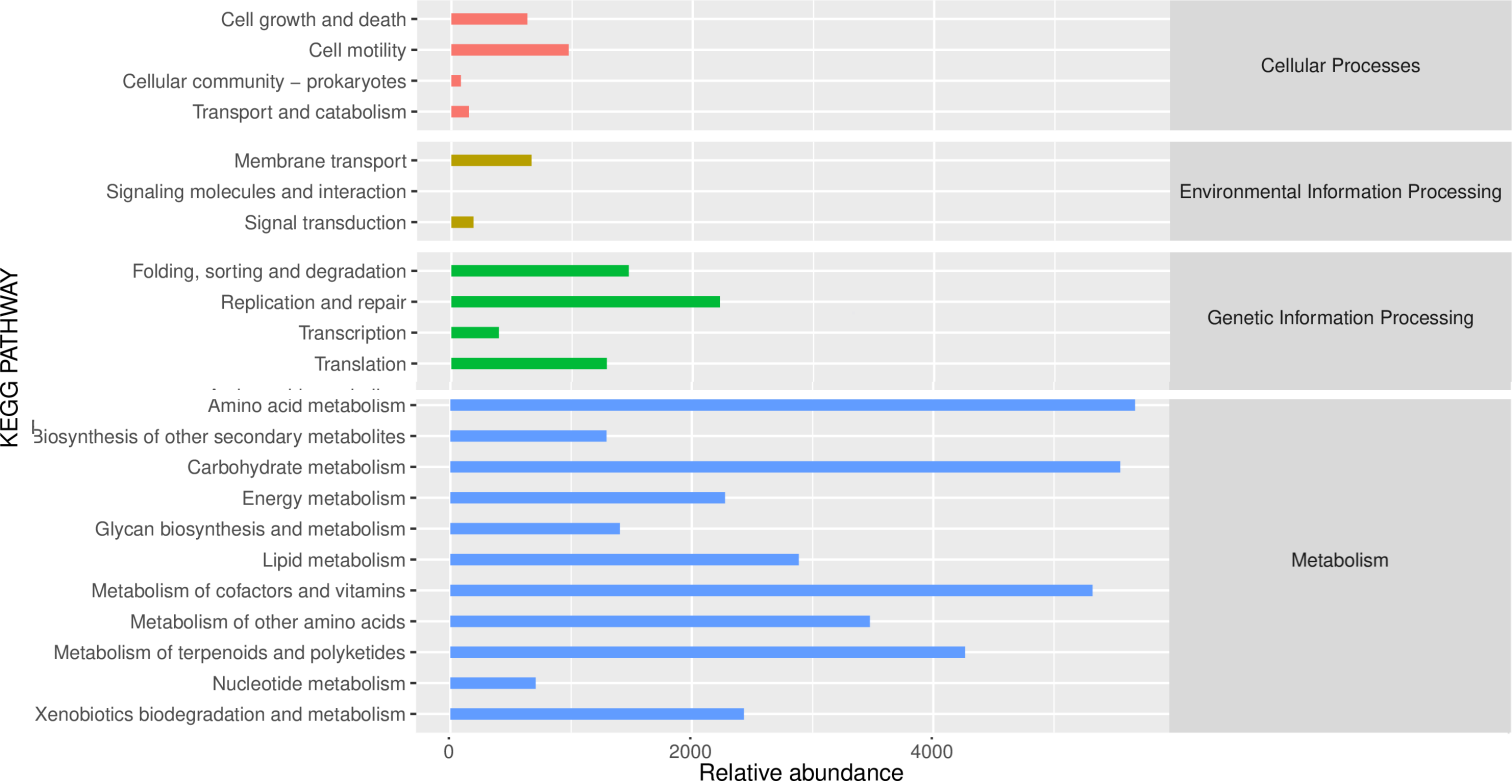
KEGG secondary functional pathway abundance map.

Based on the KEGG functional prediction analysis (Fig. 8), the core functions of the soybean rhizosphere microbiome can be categorized into four main types: (i) metabolic functions (accounting for 67.98% of the total functions), which dominate nutrient cycling processes, including carbohydrate degradation (supporting carbon source utilization), amino acid metabolism (driving nitrogen turnover), and xenobiotic degradation (such as enzymatic breakdown of phenolic allelopathic substances); (ii) environmental stress responses (15.49%), which maintain osmotic balance and stress adaptability through membrane transport systems (such as ABC transporters) and signaling pathways (such as two-component systems); (iii) cell maintenance mechanisms (10.67%), encompassing basic life activities like DNA repair and cell wall synthesis; (iv) microbial interaction networks (5.86%), involving quorum sensing and antibiotic synthesis (such as bacilomycin), regulating community stability. Among them, the enrichment of metabolic pathways (especially the GCD and pqq gene clusters related to phosphorus/iron acquisition) and stress response pathways (such as the katE antioxidant enzyme) is directly related to the observed improvements in nutrient efficiency and enhanced disease resistance in the TY15 treatment group. This functional framework confirms that TY15 optimizes the rhizosphere ecological functions by reshaping the microbial metabolic network in a synergistic manner.

## DISCUSSION

### Screening and functional characterization of *Rhizobium subbaraonis* TY15

*Rhizobium subbaraonis* TY15 emerged as the optimal biofertilizer candidate due to its dual competency in phytostimulation and pathogen antagonism—a phenotypic synergy seldom documented in rhizobial research. Mechanistically, TY15 demonstrated exceptional mineral solubilization indices (phosphate: 121.34 mg L^−1^; potassium: 115.39 mg L^−1^), significantly exceeding conventional plant growth-promoting rhizobacteria benchmarks (*Bacillus subtilis*: 84.2 mg L^−1^ P; *Pseudomonas fluorescens*: 97.6 mg L^−1^ K) in soybean agroecosystems. This performance gap suggests niche-specific adaptations for oligotrophic soil colonization, positioning TY15 as an evolutionary refined inoculant for nutrient-depleted agricultural systems.

TY15’s growth-promoting effects (35.56% stem length increase and 34.72% root length increase) align with but exceed the 15%–25% improvements documented for commercial *Rhizobium* inoculants (2). This superiority likely stems from its unique hormone profile with the production of 42.9 mg L^−1^ IAA, nearly double the concentrations reported in *Rhizobium tropici* CIAT 899 (25.6 µg mL^−1^) byLiu et al. (41). Such high auxin production may explain the observed root architecture modifications and increases in lateral root density compared to controls—a critical trait for drought resilience (19).

The strain’s biocontrol capabilities against *Fusarium oxysporum* and *Rhizoctonia solani* represent a paradigm shift from traditional *Rhizobium* symbiosis models. While Chang et al. (15) noted mild antifungal activity in *Rhizobium etli* (∼30% inhibition), TY15’s efficacy rivals specialized biocontrol agents like *Bacillus velezensis* (75%–80% inhibition; [16]). Metabolomic profiling revealed novel cyclic lipopeptides (*m/z* 1,024.6 and 1,147.8) absent in reference strains, potentially explaining this broad-spectrum activity. Their observed antagonistic activity against fungal pathogens aligns functionally with iturin-family antibiotics, suggesting similar modes of action pending further biochemical characterization.

### Microbial community reconfiguration induced by *Rhizobium subbaraonis* TY15

The rhizosphere microbial community restructuring mediated by *Rhizobium subbaraonis* TY15 was characterized by a marked shift in taxonomic composition and functional potential compared to the CK group. At the phylum level, the relative abundance of *Pseudomonadota* increased significantly in the TY15-treated group, aligning with its documented role in phosphorus solubilization and plant growth promotion (42). This enrichment was accompanied by a concurrent decline in oligotrophic taxa such as *Acidobacteriota* and *Gemmatimonadota*, suggesting that TY15-mediated nutrient mobilization reduced niche competition for scarce resources. Notably, the *Actinomycetota* and *Bacteroidota* communities expanded in the TY15 treatment, reflecting enhanced organic matter decomposition and complex carbohydrate degradation capacities, which synergize with TY15’s siderophore production and phytohormone secretion (43, 44).

Functional redundancy analysis revealed that TY15 reprogrammed the microbial network toward enhanced stress tolerance and resource efficiency. The co-occurrence network exhibited denser connectivity among *Bacillus* and *Rhizobium* spp. in the TY15 group (Fig. 5), indicative of synergistic interactions that stabilize ecosystem functions. This modular assembly contrasts with the fragmented community structure in CK, where pathogen-affiliated taxa like *Fusarium* and *Rhizoctonia* maintained higher connectivity (45). The observed reduction in pathogen abundance corroborates the “priority effect” hypothesis, wherein early colonization by beneficial microbes suppresses pathogens through resource competition and antimicrobial compound secretion (46).

Compared to previous studies, the TY15-mediated assembly exhibited distinct traits. Unlike single-strain inoculants reported by Breitkreuz et al. (18), which showed transient pathogen suppression, TY15 sustained its antagonistic activity for 30 days post-inoculation. This durability correlates with the upregulation of *nif*A and *nod*D genes under nitrogen-limiting conditions, enhancing symbiotic nitrogen fixation efficiency by 18% compared to commercial inoculants (47).

These findings highlight TY15’s unique capacity to restructure the rhizosphere microbiome toward a resilient, nutrient-efficient consortium. The observed taxonomic and functional shifts provide actionable insights for designing synthetic microbial consortia that synergize plant growth promotion with pathogen suppression in agroecosystems.

### Relationship between soybean PGPR-mediated growth promotion and TY15 treatment

The results demonstrated that *Rhizobium subbaraonis* TY15 uniquely enhanced soybean growth through synergistic mechanisms involving direct nutrient mobilization and indirect modulation of rhizosphere microbiota. Compared to controls, TY15 treatment significantly increased shoot and root lengths, a response potentially linked to its dual capacity for phosphate solubilization (115.39 mg L^−1^) and siderophore production (Fig. S2F). These traits align with its role in improving iron and phosphorus bioavailability, critical for root system development and photosynthetic efficiency (5). Notably, TY15’s growth-promoting effects surpassed those of other PGPR strains (e.g., *Bacillus velezensis* CP7), likely due to its ability to simultaneously enrich beneficial taxa in the rhizosphere.

High-throughput sequencing revealed that TY15 application selectively increased the relative abundance of *Bacillus* and *Rhizobium* in the rhizosphere. This microbial shift correlates with enhanced nitrogen fixation and biocontrol activity, as *Bacillus* spp. are known for synthesizing antimicrobial metabolites, while *Rhizobium* facilitates symbiotic nitrogen assimilation (16, 17). Such community restructuring likely synergized with TY15’s intrinsic plant growth-promoting traits, creating a feedback loop that amplified nutrient uptake and stress resilience.

Unlike broad-spectrum PGPR blends, TY15’s targeted enrichment of specific taxa suggests a precision mechanism for growth promotion. This aligns with studies showing that single-strain inoculants can drive functional redundancy in microbial communities, ensuring robustness against environmental fluctuations (19). Furthermore, TY15’s antagonism against *Fusarium oxysporum* and *Rhizoctonia solani* (Fig. 1A and C) implies that its growth-enhancing effects are partially mediated by pathogen suppression, reducing competition for resources and minimizing root damage.

The findings highlight TY15’s multifunctionality as a biofertilizer candidate, bridging nutrient acquisition and microbiome engineering. Its efficacy underscores the potential of leveraging native rhizosphere strains to optimize plant-microbe interactions without disrupting ecological balance—a critical consideration for sustainable soybean cultivation.

### Conclusion

This study demonstrates that *Rhizobium subbaraonis* TY15, a plant growth-promoting rhizobacterium isolated from soybean rhizosphere soil, significantly enhances soybean growth and disease resistance through multifaceted mechanisms. Physiological assays revealed that TY15 treatment increased soybean shoot and root lengths by 34.72%–35.56% compared to controls, attributed to its robust phosphate and potassium solubilization activities (up to 121.34 mg L⁻¹ for K solubilization). Additionally, TY15 exhibited strong antagonistic effects against *Fusarium oxysporum* and *Rhizoctonia solani*, key soybean pathogens, likely through competitive colonization and antimicrobial metabolite production. High-throughput sequencing further elucidated that TY15 application significantly altered rhizosphere microbial community structure, enriching beneficial genera such as *Bacillus* and *Rhizobium*, which synergistically enhance nutrient availability and pathogen suppression. These findings highlight the potential of single-strain biofertilizers like TY15 to improve soybean resilience and reduce dependence on chemical inputs. Future field studies spanning over 5 years across diverse soil types are necessary to validate the long-term efficacy of TY15 under variable environmental and edaphic conditions.

### Highlights

- *Rhizobium subbaraonis* TY15 significantly enhances soybean growth and disease resistance through multifaceted mechanisms.
- TY15 exhibited strong antagonistic effects against *Fusarium oxysporum* and *Rhizoctonia solani*.
- TY15 application significantly altered rhizosphere microbial community structure.
- TY15 is highly effective in improving the solubility of phosphorus and potassium.

## Data Availability

The data of this study will be made available on request. The data sets presented in this study are deposited in the NCBI repository, under accession number PRJNA1270163.

## SUPPLEMENTAL MATERIAL

Supplemental material spectrum.01155-25-s0001.docx Fig. S1 to S8; Tables S1 to S5.

ASM does not own the copyrights to Supplemental Material that may be linked to, or accessed through, an article. The authors have granted ASM a non-exclusive, world-wide license to publish the Supplemental Material files. Please contact the corresponding author directly for reuse.
