# Supplementary material for: The application of Rhizobium subbaraonis TY15 increased soybean growth and disease resistance by modifying rhizosphere microbial communities
Source: Microbiol Spectr. 2025 Nov 14;14(1):e01155-25. doi: 10.1128/spectrum.01155-25 (PMC12772353; doi:10.1128/spectrum.01155-25)
Supplement: Supplemental material — Fig. S1 to S8; Tables S1 to S5. [file spectrum.01155-25-s0001.docx]

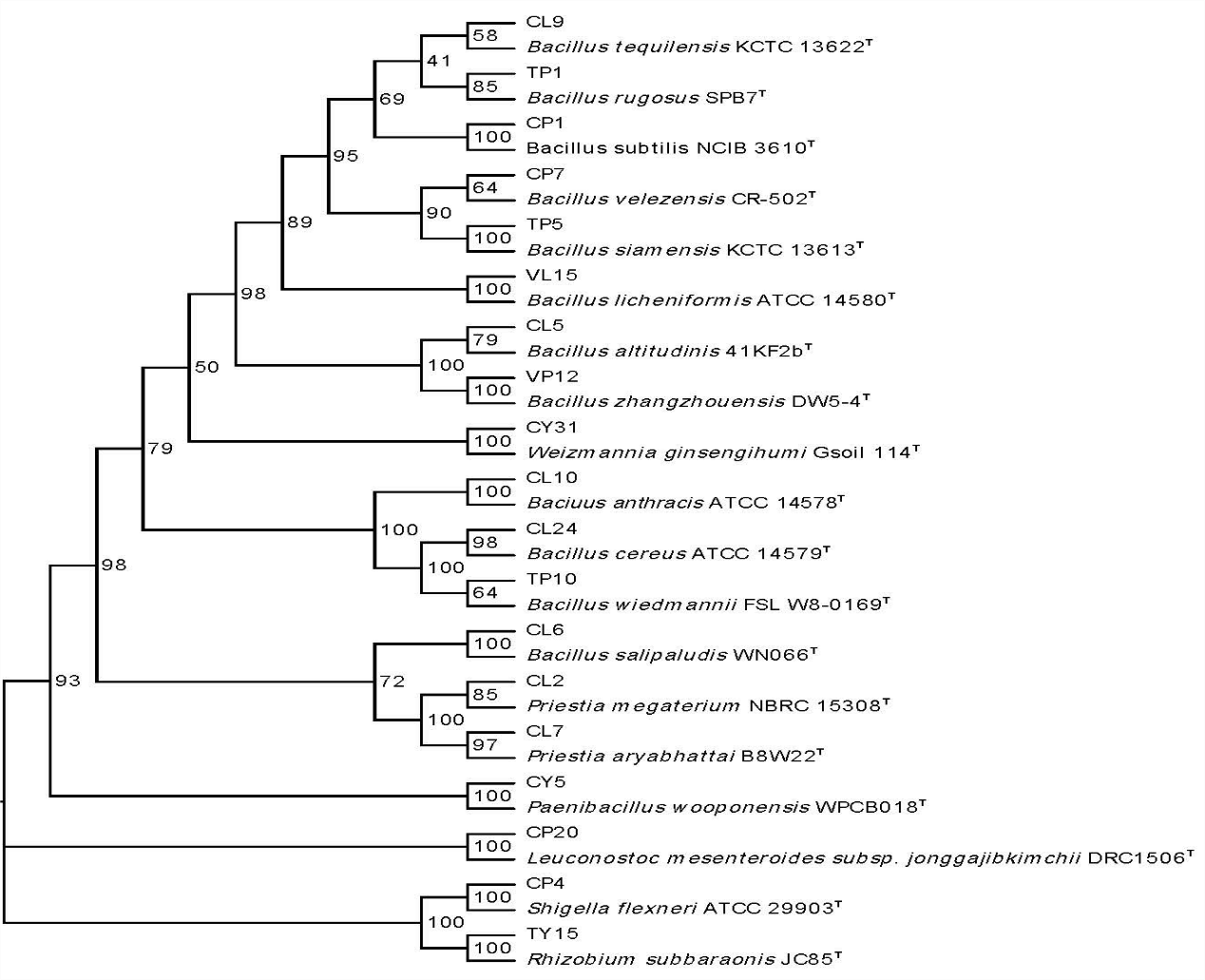


Supplementary Fig. 1. Phylogenetic tree of the 16S rRNA gene sequences of soybean rhizosphere bacteria and related strains. The tree building method is an adjacency method, where T represents the model strain, the ruler represents the nucleotide base difference of 0.05, the branch number represents the self-distance value, the number of random sampling calculations is 1000, and the brackets are the serial numbers obtained by the model strain GenBank.

Supplementary Table 1**.** Physiological and biochemical indices of soybean rhizosphere bacteria

| No. of strain | Strain  name | Ammonia productio  -n test | Cata  -lase | Methyl red test | Nitrate reductase | Gelatin liquefactio-n | V.P  test | Ureas-e | Sucrose utilizati-on |
| --- | --- | --- | --- | --- | --- | --- | --- | --- | --- |
| CL2 | *Priestia megaterium* | - | + | - | + | - | + | - | + |
| CL5 | *Bacillus altitudinis* | + | + | + | + | - | - | - | + |
| CL6 | *Bacillus salipaludis* | + | + | + | + | + | - | - | + |
| CL7 | *Priestia aryabhattai* | - | + | + | + | - | - | - | - |
| CL9 | *Bacillus tequilensis* | + | + | + | + | + | - | - | - |
| CL10 | *Bacillus anthracis* | + | + | - | + | - | - | - | - |
| CL24 | *Bacillus cereus* | + | + | + | + | - | - | - | - |
| CP1 | *Bacillus subtilis* | + | + | + | + | + | - | - | + |
| CP4 | *Shigella flexneri* | - | + | + | + | + | - | - | + |
| CP7 | *Bacillus velezensis* | + | + | + | + | - | - | - | + |
| CP20 | *Leuconostoc mesenteroides subsp. Jonggajibkimchii* | - | + | - | - | + | + | - | + |
| CY5 | *Paenibacillus wooponensis* | + | - | - | - | + | + | - | + |
| CY31 | *Weizmannia ginsengihumi Gsoil* | + | + | + | + | - | + | - | - |
| TY10 | *Bacillus wiedmannii* | + | - | - | - | - | - | + | - |
| TY15 | *Rhizobium subbaraonis* | + | + | + | + | + | - | + | - |
| TP1 | *Bacillus rugosus* | - | + | + | + | - | - | - | - |
| TP5 | *Bacillus siamensis* | + | + | + | + | + | + | - | - |
| VL15 | *Bacillus licheniformis* | - | + | + | + | + | + | - | - |
| VP12 | *Bacillus zhangzhouensis* | + | - | + | - | - | - | + | - |

Note: “+” indicates the test was positive, “−” shows the test was negative.

Supplementary Table 2. Growth-promoting characteristics of strains

| Strain number | Soluble P  content  (mgL^−1^) | Soluble K  content  (mgL^−1^) | IAA (mgL^−1^) |
| --- | --- | --- | --- |
| CL2 | 0 | 0 | 0 |
| CL5 | 0 | 0 | 0 |
| CL6 | 0 | 93.21 | 5.77 |
| CL7 | 0 | 57.32 | 6.74 |
| CL9 | 97.63 | 0 | 0 |
| CL10 | 3.51 | 0 | 0 |
| CL24 | 0 | 0 | 0 |
| CP1 | 0 | 115.39 | 8.05 |
| CP4 | 0 | 35.64 | 0 |
| CP7 | 69.77 | 48.34 | 5.81 |
| CP20 | 121.34 | 85.64 | 14.81 |
| CY5 | 0 | 31.68 | 0 |
| CY31 | 78.75 | 0 | 0 |
| TY10 | 0 | 0 | 11.37 |
| TY15 | 0 | 39.64 | 42.90 |
| TP1 | 0 | 0 | 0 |
| TP5 | 83.86 | 0 | 0 |
| VL15 | 52.10 | 0 | 0 |
| VP12 | 88.66 | 0 | 0 |

Supplementary Table 3. Amylase, cellulase and protease production of soybean rhizosphere bacteria

| Disease-resistance related enzyme | Strain number | *D*/mm  Dissolving ring  diameter | *d*/mm  Colony  diameter | *D*/*d* |
| --- | --- | --- | --- | --- |
|  | CK | 0.00±0.00 | 0.00±0.00 | 0.00±0.00 |
| Amylase | CL7 | 26.50 ±0.23 | 19.98 ±0.32 | 1.33 ±0.02 bc |
|  | CL24 | 38.14 ±5.38 | 28.41 ±1.91 | 1.34 ±0.12 bc |
|  | CP7 | 31.31 ±5.28 | 16.46 ±2.22 | 1.93 ±0.44 a |
|  | TP1 | 21.14 ±0.67 | 12.74 ±0.72 | 1.66 ±0.04 ab |
|  | TY10 | 36.74 ±3.87 | 24.40 ±1.84 | 1.51 ±0.15 bc |
|  | VP12 | 25.37 ±1.06 | 19.90 ±1.28 | 1.28 ±0.05 c |
| Cellulase | CL2 | 33.25 ±5.11 | 15.50 ±0.71 | 2.15 ±0.30 cde |
|  | CL5 | 22.55 ±4.17 | 6.65 ±2.52 | 3.65 ±1.08 ab |
|  | CL6 | 37.38 ±5.66 | 22.59 ±1.07 | 1.66 ±0.26 de |
|  | CL9 | 31.50 ±7.44 | 13.13 ±5.11 | 2.63 ±1.17 bcd |
|  | CL24 | 14.67 ±4.80 | 3.37 ±0.08 | 4.34 ±1.39 a |
|  | CP1 | 36.27 ±3.59 | 19.44 ±0.96 | 1.86 ±0.09 de |
|  | CP4 | 32.74 ±5.63 | 14.17 ±0.23 | 2.31 ±0.41 cde |
|  | CP7 | 30.17 ±2.30 | 9.70 ±2.70 | 3.27 ±0.97 abc |
|  | CP20 | 32.70 ±7.10 | 16.33 ±2.28 | 1.99 ±0.22 de |
|  | CY5 | 38.88 ±3.47 | 31.72 ±8.07 | 1.27 ±0.26 e |
|  | CY31 | 7.03 ±0.67 | 2.57 ±0.25 | 2.76 ±0.49 bcd |
|  | TP1 | 19.72 ±3.43 | 6.19 ±1.04 | 3.19 ±0.30 abc |
|  | TP5 | 37.12 ±5.63 | 29.09 ±5.01 | 1.28 ±0.16 e |
|  | TY10 | 14.94 ±0.64 | 5.72 ±0.78 | 2.64 ±0.37 bcd |
|  | TY15 | 6.24 ±1.65 | 3.32 ±0.82 | 1.88 ±0.04 de |
|  | VL15 | 21.19 ±3.74 | 6.32 ±0.44 | 3.34 ±0.39 abc |
|  | VP12 | 25.88 ±5.49 | 6.28 ±2.18 | 4.25 ±0.57 a |
| Protease | CL2 | 28.38± 4.21 | 15.98 ±1.81 | 1.78 ±0.19 bcdef |
|  | CL5 | 24.89±0.65 | 12.09 ±3.43 | 2.16 ±0.54 abc |
|  | CL6 | 36.12±12.50 | 20.57 ±9.08 | 1.80 ±0.15 abcdef |
|  | CL7 | 23.97±2.14 | 12.97 ±1.60 | 1.86 ±0.15 abcde |
|  | CL9 | 31.46±5.28 | 13.72± 0.38 | 2.30 ±0.40 ab |
|  | CL24 | 34.28±5.50 | 28.56 ±3.89 | 1.21 ±0.23 f |
|  | CP1 | 35.04±5.98 | 20.07±3.00 | 1.74 ±0.06 bcdef |
|  | CP4 | 33.23±4.45 | 18.24 ±0.78 | 1.83 ±0.33 abcde |
|  | CP7 | 34.59±1.43 | 24.53±4.00 | 1.43 ±0.16 ef |
|  | CP20 | 32.41±3.00 | 15.28±1.31 | 2.12 ±0.05 abcd |
|  | CY5 | 35.59±3.60 | 21.28±2.71 | 1.68 ±0.11 cdef |
|  | CY31 | 12.48±5.91 | 7.14 ±1.10 | 1.75 ±0.75 bcdef |
|  | TP1 | 20.03±1.10 | 12.82±0.96 | 1.57 ±0.10 cdef |
|  | TP5 | 32.95±3.72 | 20.35±1.09 | 1.62 ±0.10 cdef |
|  | TY10 | 34.10±4.46 | 22.48±7.21 | 1.62 ±0.58 cdef |
|  | TY15 | 24.28±2.24 | 10.29±1.84 | 2.38 ±0.21 a |
|  | VL15 | 22.43±4.31 | 14.77 ±3.29 | 1.53 ±0.19 def |
|  | VP12 | 31.62±4.31 | 20.13 ±4.40 | 1.59 ±0.15 cdef |


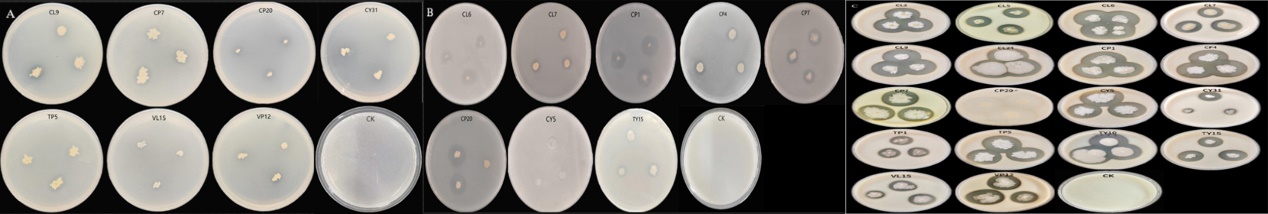


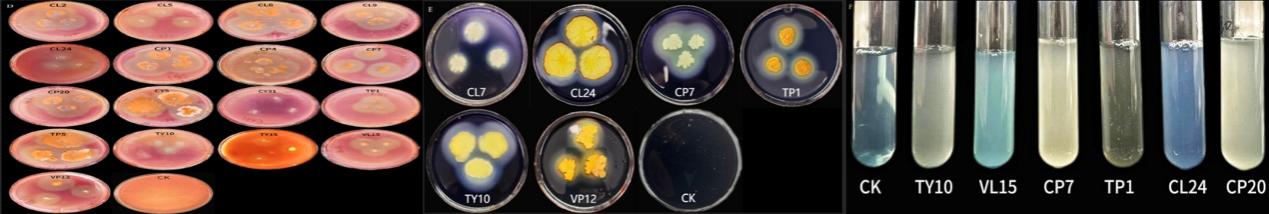


Supplementary Fig. 2. Growth-promoting characteristics of related strains: P, K solubilization (A&B), proteinase production (C), cellulase production (D), amylase production (E) and siderophore production (F).

Supplementary Table 4. Growth-promoting characteristics of strains

| Strain number | Chitinase  content (mgL^-1.^) | ACC deaminase content (mgL^-1.^) |
| --- | --- | --- |
| CL2 | 0 | 0 |
| CL5 | 0 | 0 |
| CL6 | 0 | 0 |
| CL7 | 0 | 0 |
| CL9 | 0 | 0 |
| CL10 | 0 | 0 |
| CL24 | 0 | 0 |
| CP1 | 0 | 0 |
| CP4 | 0 | 2.83 |
| CP7 | 7.98 | 11.54 |
| CP20 | 4.01 | 0 |
| CY5 | 0 | 4.74 |
| CY31 | 3.33 | 1.31 |
| TY10 | 0 | 0 |
| TY15 | 5.05 | 6.64 |
| TP1 | 0 | 0 |
| TP5 | 0 | 0 |
| VL15 | 0 | 0 |
| VP12 | 0 | 0 |


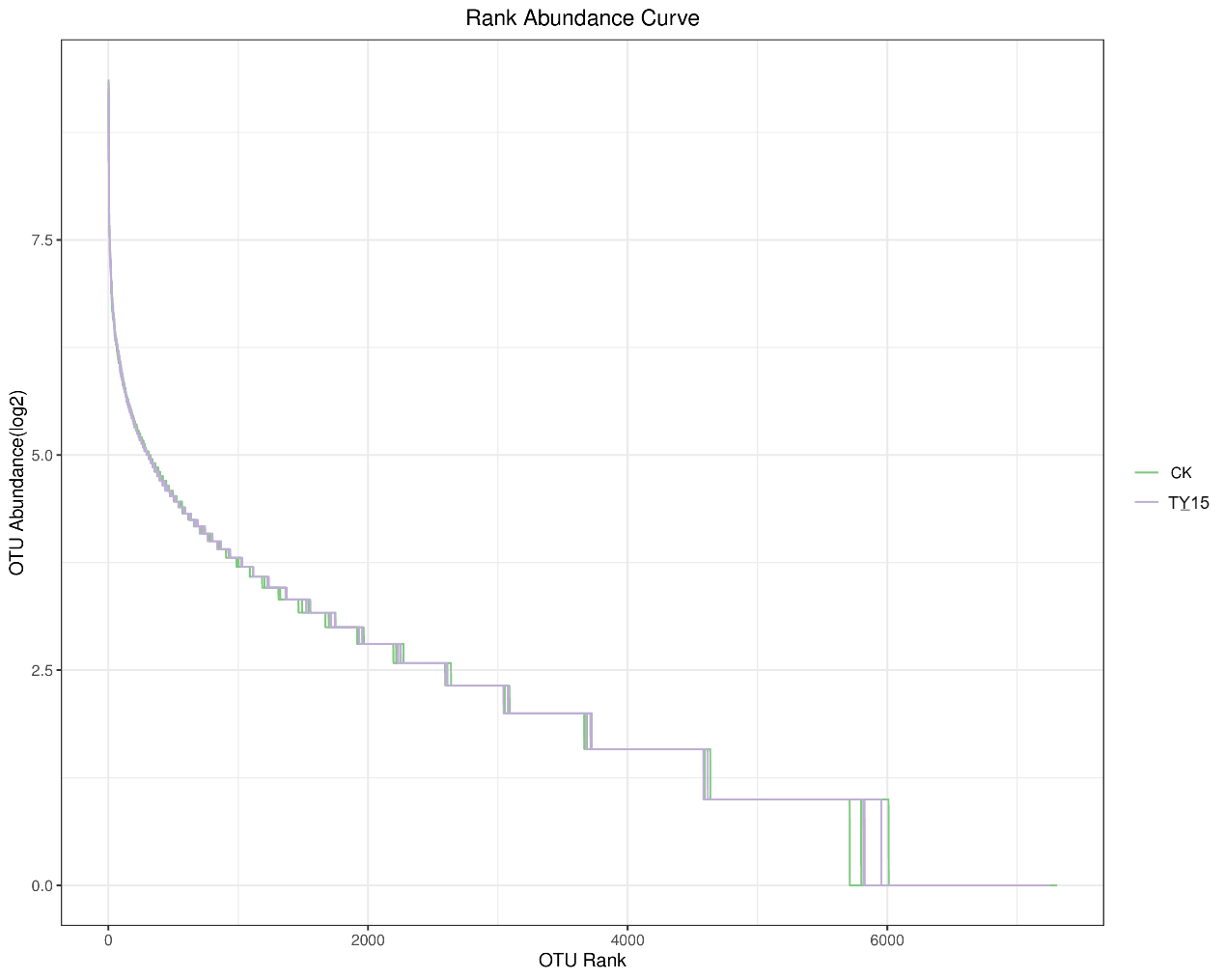


Supplementary Fig. 3. Abundance grade graph


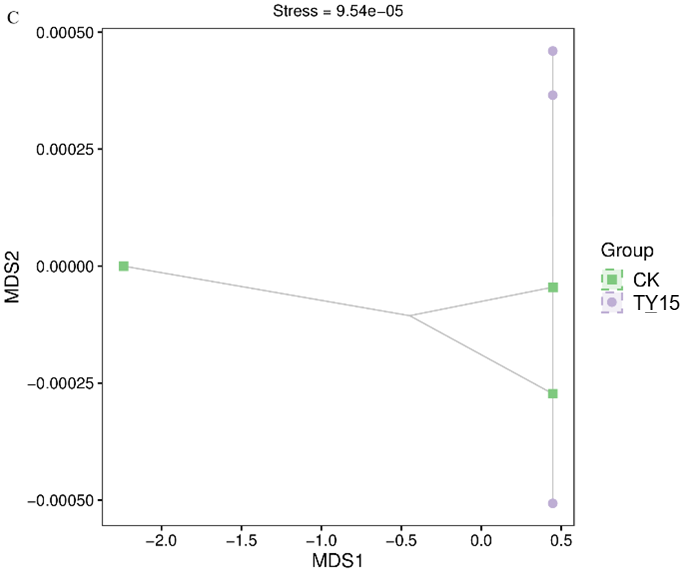


Supplementary Fig. 4. NMDS two-dimensional sequencing diagram

Supplementary Table 5. Alpha diversity index statistics [no significant differences (p>0.05)]

| **Sample** | **Chao1** | **Simpson** | **Shannon** | **Pielou_e** | **Observed_species** | **Faith_pd** | **Goods_coverage** |
| --- | --- | --- | --- | --- | --- | --- | --- |
| **CK** | 4755.8133 ±298.4622 | 0.9983 ±0.00014 | 10.9330 ±0.06847 | 0.8989 ±0.00059 | 4588.8667 ±244.29117 | 266.2853 ±12.98372 | 0.9913 ±0.00175 |
| **V66** | 5019.5733 ±756.6953 | 0.9991 ±0.00005 | 11.2381 ±0.14753 | 0.9185 ±0.00301 | 4853.6333 ±632.02908 | 267.9167 ±22.35126 | 0.9917 ±0.00476 |


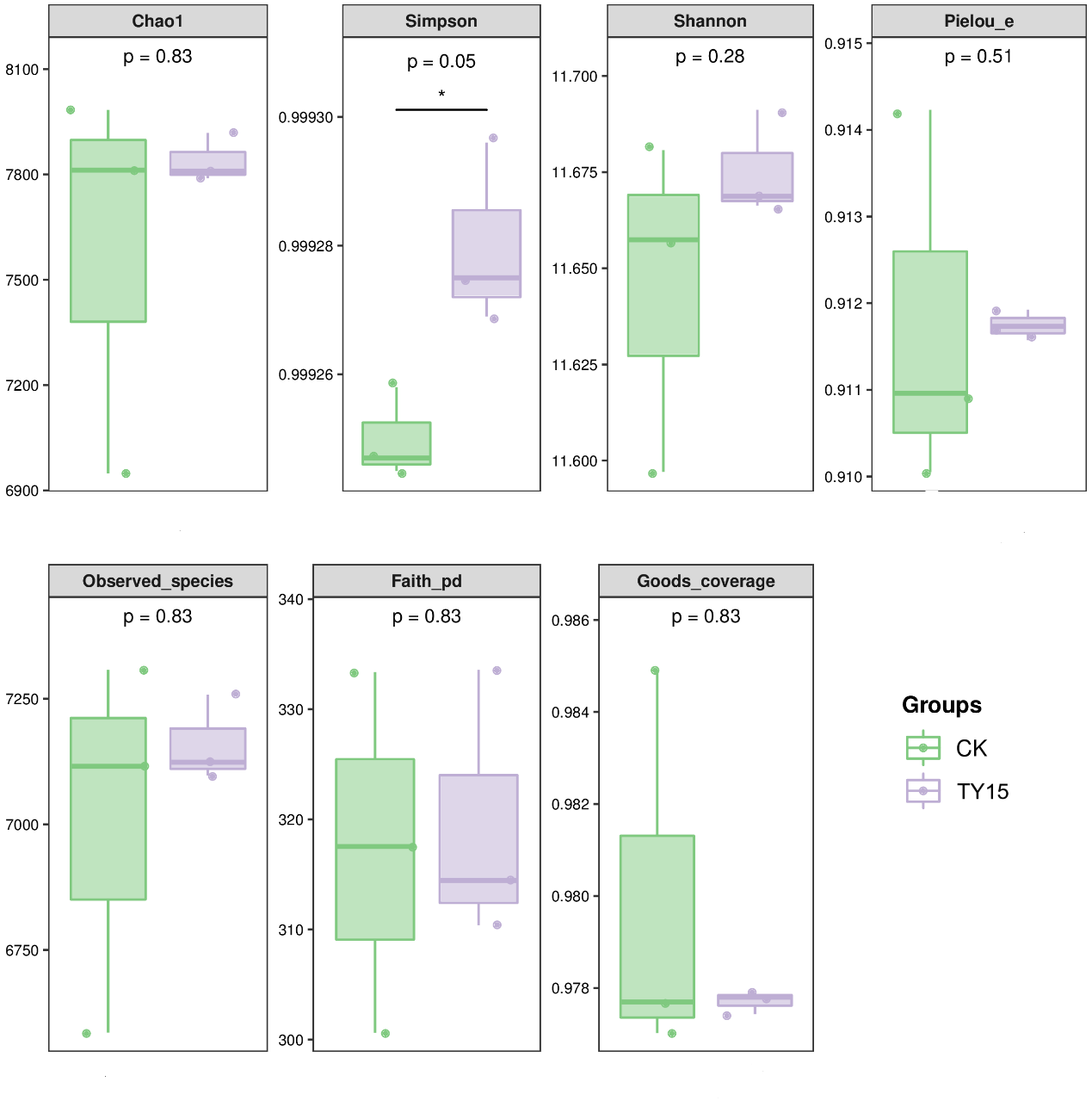


Supplementary Fig. 5. Grouped boxplot of the alpha diversity index


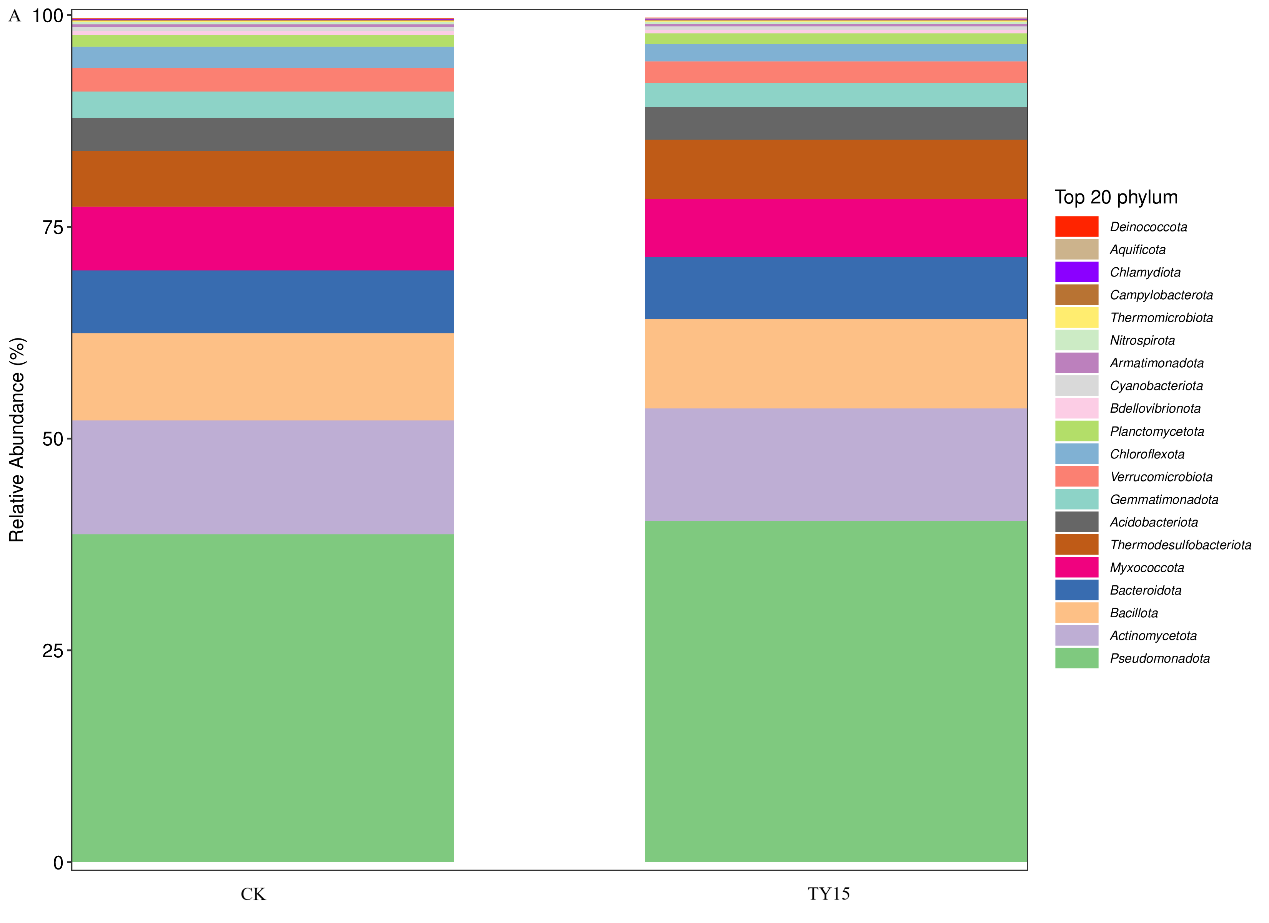


Supplementary Fig. 6. Histogram of the species composition of horizontal species


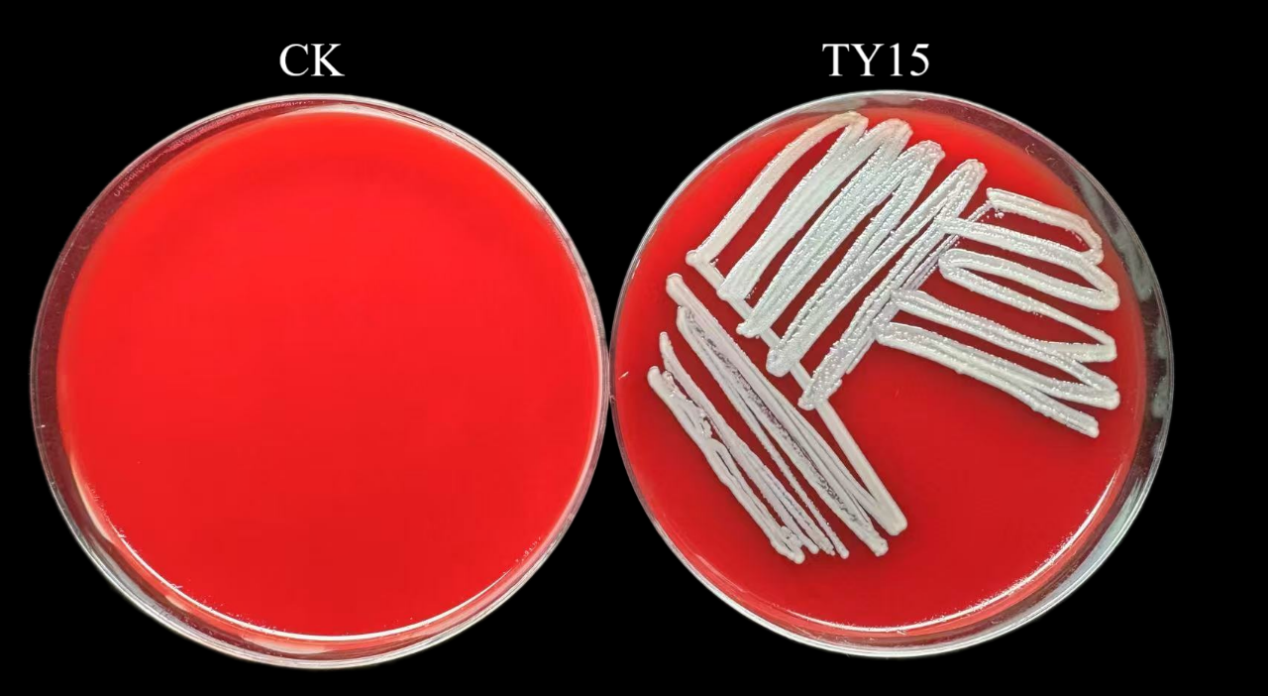


Supplementary Fig.7. Comparative Growth and Hemolytic Phenotype Analysis of Control (CK) versus TY15 Strain on Blood Agar Medium


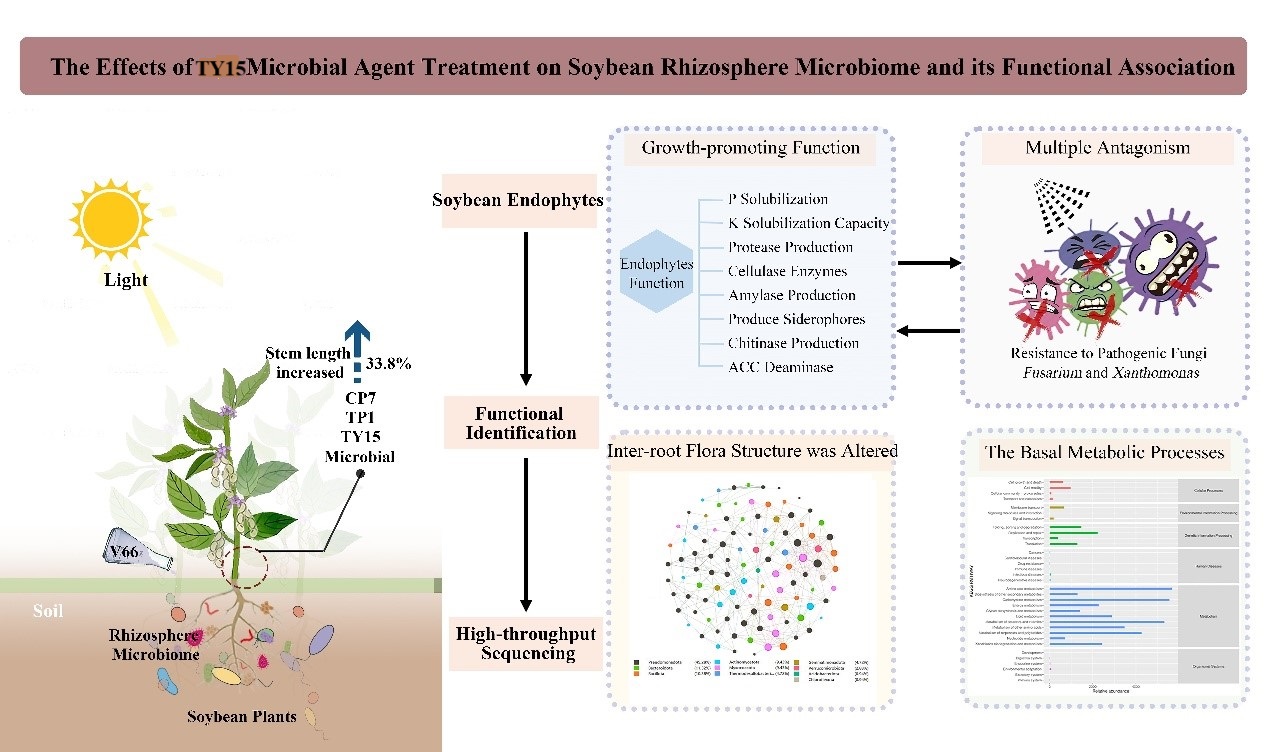


Supplementary Fig.8. Abstract graphic
